# Magnetic mesoporous silica nanoparticles modified by phosphonate functionalized ionic liquid for selective enrichment of phosphopeptides†

**DOI:** 10.1039/d2ra04609a

**Published:** 2022-09-21

**Authors:** Yufei Jiang, Weida Liang, Binbin Wang, Quanshou Feng, Chenglong Xia, Qiyao Wang, Yinghua Yan, Lingling Zhao, Wei Cui, Hongze Liang

**Affiliations:** Key Laboratory of Advanced Mass Spectrometry and Molecular Analysis of Zhejiang Province, School of Materials Science and Chemical Engineering, Ningbo University Ningbo 315211 China lianghongze@nbu.edu.cn; Ningbo Key Laboratory of Behaviour Neuroscience, Zhejiang Province Key Laboratory of Pathophysiology, School of Medicine, Ningbo University Ningbo 315211 China cuiwei@nbu.edu.cn

## Abstract

In this study, new magnetic nanoparticles (denotated as Fe_3_O_4_@mSiO_2_-PFIL-Ti^4+^) have been prepared by immobilizing titanium ions with phosphonate functionalized ionic liquid (PFIL) on the wall of core–shell structured mesoporous nanomaterials. The resulting nanoparticles possess large specific surface area, strong hydrophilicity and fast magnetic response. The composites can capture traces of phosphopeptides from the tryptic β-casein digest (0.08 fmol), a digest mixture of β-casein and BSA (1 : 10 000, molar ratio) as well as a blend of β-casein digest and a great quantity of phosphorylated protein (β-casein) and non-phosphorylated protein (BSA) (1 : 2000 : 2000, mass ratio), respectively, showing excellent sensitivity, selectivity and size exclusion ability. Additionally, Fe_3_O_4_@mSiO_2_-PFIL-Ti^4+^ shows excellent steadiness and can be reused at least 12 times. Moreover, this material was successfully applied to enrich endogenous phosphopeptides from complex bio-samples, including human saliva and serum.

## Abstract 2

A new magnetic IMAC core–shell nanomaterial (Fe_3_O_4_@mSiO_2_-PFIL-Ti^4+^) modified with phosphonate functionalized ionic liquid (PFIL) has been prepared and exhibits excellent specific enrichment of phosphopeptides.

## Graphical abstract

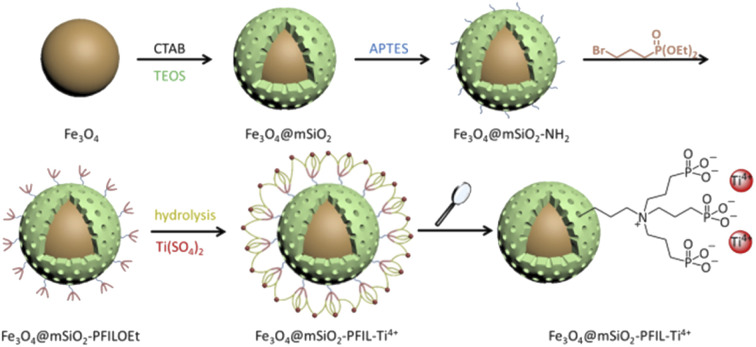

## Introduction

Protein phosphorylation is widely recognized as a principal post-translational modification. In most cases, it plays an important part in biological processes, such as cell growth and division, as well as intercellular signal transduction.^1–5^ Abnormal phosphorylation would incur ailments and even serious diseases. Insight into the role of protein phosphorylation is of great importance for grasping the pathological mechanisms and thus helps diagnosis and drug development. Over the past decades, with the aid of mass spectrometric techniques, phosphoproteomics has developed quickly. However, reliable information from mass spectrometry (MS) is highly dependent on some factors. Suppression from highly abundant proteins and nonphosphopeptides and low ionization efficiency of phosphopeptides will deteriorate the quality of MS spectra and may lead to the loss of some signals of exceedingly low-abundant phosphopeptides in complex bio-samples. Therefore, in order to eliminate interferences and then enhance signal detection ability to realize identification of phosphorylation efficiently, treatment of the complex samples with highly specific and sensitive adsorbents prior to MS is necessary.^6^

So far, various strategies have been exploited for phosphopeptides enrichment.^7–10^ Among them, the most important methodologies are the immobilized metal ion affinity chromatography (IMAC) based on the affinity of metal ions with the phosphate groups from phosphopeptides,^4,11,12^ and the metal oxide affinity chromatography (MOAC) based on the affinity of metal oxides with the phosphate groups.^13–15^ The structures and extraction properties of IMAC materials are more tunable due to free combination of the building blocks including ligands, linkers, and metal ions. The space linkers are flexible and can be adjusted to reduce the steric hindrance when approaching to phopshopeptides. Chelating ligands can strongly immobilize metal ions and thus the leaching of metals can be dramatically reduced. These advantages may render IMAC materials suitable to distinguish target molecules from the rest in complicated samples.

The nature of IMAC adsorbent substrates is also an important factor affecting the extraction performance. Nanomaterials and mesoporous materials have been extensively studied for the application in biosciences.^16–18^ Besides, magnetic materials have also drawn much attention due to the rapid magnetic responsiveness that can be used to accelerate separation process.^19–23^ Separation and enrichment by nonmagnetic materials is a time-consuming procedure usually including sample loading, centrifuging, washing and eluting; thereby tendency of contamination and loss of target molecules in low abundance is increased.^24^ Magnetic nanomaterials and mesoporous materials with suitable surface modification can enrich phosphopeptides and accelerate the separation process in short time with the aid of external magnetic field.

We recently reported the synthesis of a new IMAC nanoparticles (G@mSiO_2_-PFIL-M^*n*+^), in which PFIL (phosphonate-functionalized ionic liquid) was firstly introduced as a chelating ligand.^25^ Excellent sensitivity and specificity were observed in phosphopeptides enrichment. Herein, we report the fabrication of magnetic core–shell adsorbent material that was modified by PFIL to achieve fast separation. The prepared magnetic IMAC material (denoted as Fe_3_O_4_@mSiO_2_-PFIL-Ti^4+^) exhibited excellent specificity, sensitivity, and reusability. Moreover, this adsorbent also showed prominent size-exclusion effect when it was employed to capture phosphopeptides from the blend of β-casein digest and proteins. The ability of the freshly prepared Fe_3_O_4_@mSiO_2_-PFIL-Ti^4+^ nanoparticles to capture endogenous phosphopeptides from bio-samples such as saliva and serum was further studied and compared with commercial PurMag Si–TiO_2_.

## Results and discussion

### Preparation of Fe_3_O_4_@mSiO_2_-PFIL-Ti^4+^

**Scheme 1:**
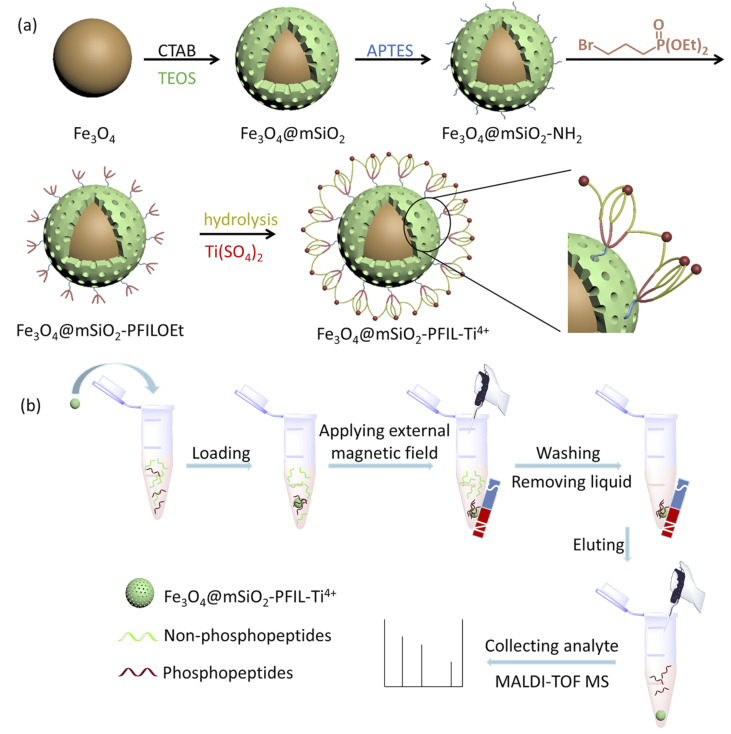
(a) Synthesis diagram of Fe_3_O_4_@mSiO_2_-PFIL-Ti^4+^ magnetic nanoparticles; (b) steps of phosphopeptides enrichment from samples by magnetic separation of Fe_3_O_4_@mSiO_2_-PFIL-Ti^4+^ magnetic nanoparticles.

The preparation procedure of the material is exhibited in Scheme 1a. Magnetic Fe_3_O_4_ nanoparticles were coated with mesoporous silica by hydrolyzation of TEOS in the presence of CTAB. The resulting Fe_3_O_4_@mSiO_2_ was aminated by APTES to form Fe_3_O_4_@mSiO_2_-NH_2_, which was further quaternized with diethyl 3-bromopropylphosphonate. The obtained Fe_3_O_4_@mSiO_2_-PFILOEt was hydrolysed with Si(CH_3_)_3_Br followed by neutralization with NaOH solution and immobilization with Ti(SO_4_)_2_ to afford the final magnetic nanoparticles Fe_3_O_4_@mSiO_2_-PFIL-Ti^4+^.

### Characterization of Fe_3_O_4_@mSiO_2_-PFIL-Ti^4+^

**Fig. 1:**
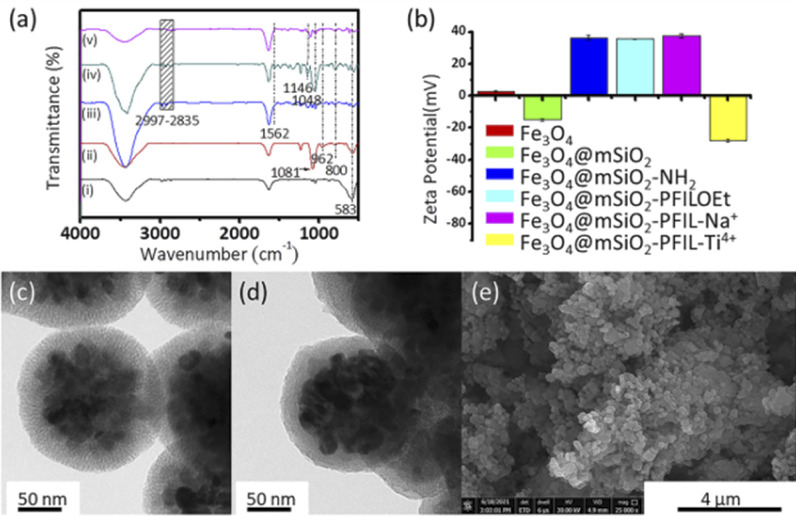
Structural characterizations of nanoparticles. (a) FT-IR spectra of Fe_3_O_4_ (i), Fe_3_O_4_@mSiO_2_ (ii), Fe_3_O_4_@mSiO_2_-NH_2_ (iii), Fe_3_O_4_@mSiO_2_-PFILOEt (iv), and Fe_3_O_4_@mSiO_2_-PFIL-Ti^4+^ (v). (b) Zeta potentials of Fe_3_O_4_, Fe_3_O_4_@mSiO_2_, Fe_3_O_4_@mSiO_2_-NH_2_, Fe_3_O_4_@mSiO_2_-PFILOEt, Fe_3_O_4_@mSiO_2_-PFIL-Na^+^, and Fe_3_O_4_@mSiO_2_-PFIL-Ti^4+^. TEM images of (c) Fe_3_O_4_@mSiO_2_ and (d) Fe_3_O_4_@mSiO_2_-PFIL-Ti^4+^. (e) SEM image of Fe_3_O_4_@mSiO_2_-PFIL-Ti^4+^.

The characterization of the fabricated Fe_3_O_4_@mSiO_2_-PFIL-Ti^4+^ was performed. IR absorption peak at 583 cm^−1^ was discerned on all five nanoparticles in Fig. 1a, complying with the vibration of Fe–O bond from the magnetite phase. Peaks appeared at 3447 cm^−1^ and 1635 cm^−1^ are attributed to the O–H stretching and H–O–H bending vibrations from absorbed water, respectively. For Fe_3_O_4_@mSiO_2_, the absorption bands observed at 962 cm^−1^ correspond to the bending vibration of Si–OH bond, while the absorption bands observed at 1081 and 800 cm^−1^ pertain to stretching vibration of the Si–O–Si bond. Methylene stretching vibrations of propyl group (2997–2835 cm^−1^), and bending vibration of N–H (1562 cm^−1^) demonstrate that aminopropyl group was successfully grafted onto the surface of the magnetic nanoparticles. The absorption bands at 1146 and 1046 cm^−1^, attributed to the stretching vibration of P=O and P–O, respectively, can be observed for Fe_3_O_4_@mSiO_2_-PFILOEt and Fe_3_O_4_@mSiO_2_-PFIL-Ti^4+^. All these facts proved the successful synthesis of Fe_3_O_4_@mSiO_2_-PFIL-Ti^4+^.

To further prove the successful preparation of these magnetic nanoparticles, zeta potential was measured for these nanospheres (Fig. 1b). After amino-functionalization, the zeta potential of Fe_3_O_4_@mSiO_2_-NH_2_ nanoparticles was changed from −15.0 to 36.4 mV. Although the surface zeta potential of Fe_3_O_4_@mSiO_2_-PFILOEt nanoparticles (35.6 mV) decrease slightly, it is still positive after quaternization as a result of the positive charge nature of the ionic liquid skeleton.^25^ Neutralization with sodium hydroxide made the potential of Fe_3_O_4_@mSiO_2_-PFIL-Na^+^ slightly changed to 37.4 mV. The immobilization of Ti^4+^ led to a sharp reduction in the zeta potential of Fe_3_O_4_@mSiO_2_-PFIL-Ti^4+^ to −27.9 mV.

Transmission electron microscopy (TEM) and scanning electron microscopy (SEM) were employed to monitor the morphological change of magnetic spheres. From Fig. 1c, the TEM image indicates that magnetic materials coated with mesoporous silica were prepared successfully. The surface of Fe_3_O_4_ nanoparticles is coated with a mesoporous SiO_2_ layer, and the size increases from about 100 nm to about 200 nm. The radialized channels can be seen in Fig. 1c, proving the triumphant coating of mesoporous SiO_2_. It can be observed from the change of channels in Fig. 1c and d that the IMAC material has been functionally modified on the inner wall of channels that is beneficial to the loading of more metal ions. The SEM image (Fig. 1e) of Fe_3_O_4_@mSiO_2_-PFIL-Ti^4+^ shows the uniform size while the energy dispersive X-ray analysis (EDX) result (Fig. S1, ESI†) also confirmed the successful preparation of the magnetic IMAC material.

In order to get insight into the compositions of Fe_3_O_4_@mSiO_2_-PFIL-Ti^4+^, it was further analyzed by X-ray photoelectron spectrometry (XPS). As demonstrated in Fig. S2 (ESI†), the element peaks of Fe 2p, O 1s, Ti 2p, N 1s, C 1s, P 2p, Si 2p and Fe 3p could be observed in the spectrum, implying the successful synthesis of Fe_3_O_4_@mSiO_2_-PFIL-Ti^4+^. In addition, inductively coupled plasma optical emission spectroscopy (ICP-OES) analysis was carried out to provide the quantity of Ti (7.47 μg mg^−1^) immobilized on Fe_3_O_4_@mSiO_2_-PFIL-Ti^4+^.

**Fig. 2:**
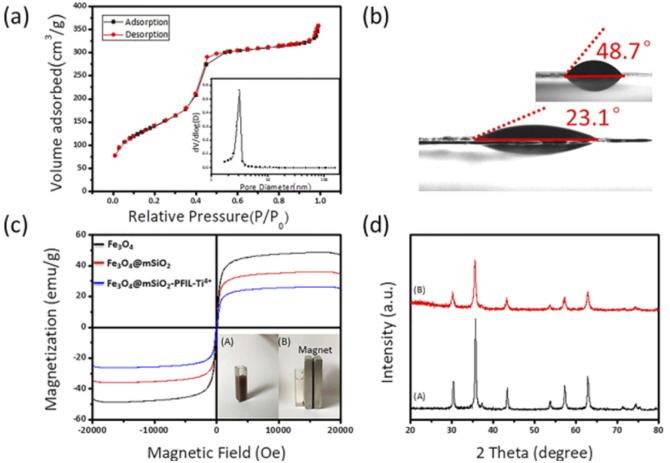
Structural characterizations of nanoparticles. (a) N_2_ adsorption–desorption isotherms and pore size distribution (inset) of Fe_3_O_4_@mSiO_2_-PFIL-Ti^4+^. (b) Water contact angle of Fe_3_O_4_@mSiO_2_-PFIL-Ti^4+^ (inset, Fe_3_O_4_@mSiO_2_-PFILOEt). (c) Magnetic hysteresis curves of the synthesized Fe_3_O_4_, Fe_3_O_4_@mSiO_2_ and Fe_3_O_4_@mSiO_2_-PFIL-Ti^4+^ nanoparticles and the photos of dispersions of Fe_3_O_4_@mSiO_2_-PFIL-Ti^4+^ nanoparticles: (A) before and (B) after exerting an applied magnetic field for 15 s. (d) XRD pattern of (A) Fe_3_O_4_ and (B) Fe_3_O_4_@mSiO_2_-PFIL-Ti^4+^.

The surface area, pore size and pore volume of the Fe_3_O_4_@mSiO_2_-PFIL-Ti^4+^ nanoparticles were determined by N_2_ adsorption–desorption measurements (Fig. 2a). The Brunauer–Emmet–Teller (BET) surface area and total pore volume are 509.77 m^2^ g^−1^ and 0.55 cm^3^ g^−1^, respectively. The pore size distribution calculated by Barrett–Joyner–Halenda (BJH) model shows the average pore size is about 3.13 nm which is similar to the previous results of our research group.^25^ Such a large pore volume and narrow pore size can increase the surface area of the nanoparticles, thereby enhancing the enrichment capacity and decreasing interferences of large biomolecules. The adsorption capacity of Fe_3_O_4_@mSiO_2_-PFIL-Ti^4+^ nanoparticles was evaluated by setting pyridoxal 5′-phosphate as the pattern, which was calculated to be 9.9 mg g^−1^.

Water contact angle embodies the interface characteristics from solid material surface to water. Fig. 2b vividly shows that after diethyl phosphonate precursor Fe_3_O_4_@mSiO_2_-PFILOEt was hydrolysed, the water contact angle of the prepared Fe_3_O_4_@mSiO_2_-PFIL-Ti^4+^, was reduced from 48.7° to 23.1°. Compared with previous studies, hydrophilicity was enhanced.^25^ Although the spacer arms and diethyl phosphate groups would enhance the hydrophobicity of the material, the cationic skeleton introduced into the designed material can greatly improve its hydrophilicity, and lead to a large decrease of the water contact angle of the final nanoparticles, proving the rationality of the design strategy.

The magnetic properties of Fe_3_O_4_, Fe_3_O_4_@mSiO_2_ and Fe_3_O_4_@mSiO_2_-PFIL-Ti^4+^ nanoparticles were measured by vibrating sample magnetometer (VSM). As shown in Fig. 2c, saturation magnetization of Fe_3_O_4_, Fe_3_O_4_@mSiO_2_ and Fe_3_O_4_@mSiO_2_-PFIL-Ti^4+^ nanoparticles are 48.5, 35.9, and 26.2 emu g^−1^, respectively. Although the saturation magnetization of this final product is reduced due to the mesoporous SiO_2_ layer and the subsequent modification, it can be seen that under the action of external magnetic field, Fe_3_O_4_@mSiO_2_-PFIL-Ti^4+^ nanoparticles can still be gathered rapidly from the suspension within 15 s.

Furthermore, the crystal structures of the Fe_3_O_4_ and Fe_3_O_4_@mSiO_2_-PFIL-Ti^4+^ nanospheres were identified by X-ray diffraction (XRD). As shown in Fig. 2d, the diffraction peaks of Fe_3_O_4_ nanoparticles with 2*θ* at 30.4°, 35.6°, 43.3°, 57.3°, and 62.8° were observed, proving that the structure of the nanoparticles is cubic spinel structure.^26^ The diffraction peaks of Fe_3_O_4_@mSiO_2_-PFIL-Ti^4+^ nanoparticles can also be viewed in Fig. 2d, which completely matches the diffraction pattern of Fe_3_O_4_ nanoparticles. It can be fully proved that the crystalline type has not been varied after a series of coating and molecular modification. This result is consistent with the properties of magnetic measurement. Through all these characterizations, the novel IMAC magnetic nanoparticles were proved to be successfully prepared, with high hydrophilicity and excellent magnetic response property.

### Optimization of loading and elution buffers

The visual enrichment procedure is shown in Scheme 1b. Loading and elution buffers have great effects on phosphopeptides enrichment. Therefore, optimizing buffers was essential. Firstly, the effect of acidity was investigated by varying the volume ratio of ACN and TFA in loading buffer (50% ACN containing TFA, from 0.1%, 0.2%, 0.5%, 1%, 2% to 3%, v/v) on specifically capturing phosphopeptides from the tryptic digest mixture of β-casein and BSA (1 : 100, molar ratio). The acquired phosphopeptides were eluted with 3% (v/v) of ammonia solution (diluted by H_2_O). In Fig. S3 (ESI†), when loading buffer consisting of 50% ACN and 1% TFA was used, Fe_3_O_4_@mSiO_2_-PFIL-Ti^4+^ nanoparticles show excellent specific enrichment to phosphopeptides and 4 phosphopeptides (*m*/*z* 2061, 2556, 2966, and 3122) were observed in Fig. S3d (ESI†).

Keeping the loading buffer fixed at 50% ACN and 1% TFA, the influence of the concentration of NH_4_OH was investigated. The trapped phosphopeptides were eluted using three concentrations of NH_4_OH diluted with water. Fig. S4 (ESI†) indicated that strong peaks of three phosphopeptides (*m*/*z* 2061, 2556, and 3122) with clean background were found by using ammonia solution (concentrated ammonia solution diluted with deionized water, 5%, v/v) as elution buffer. Thus, this concentration of NH_4_OH solution was employed for the following experiments.

All detailed information of the phosphopeptides enriched by Fe_3_O_4_@mSiO_2_-PFIL-Ti^4+^ is listed in ESI (Table S1†).

### Enrichment of phosphopeptides from the standard proteins

**Fig. 3:**
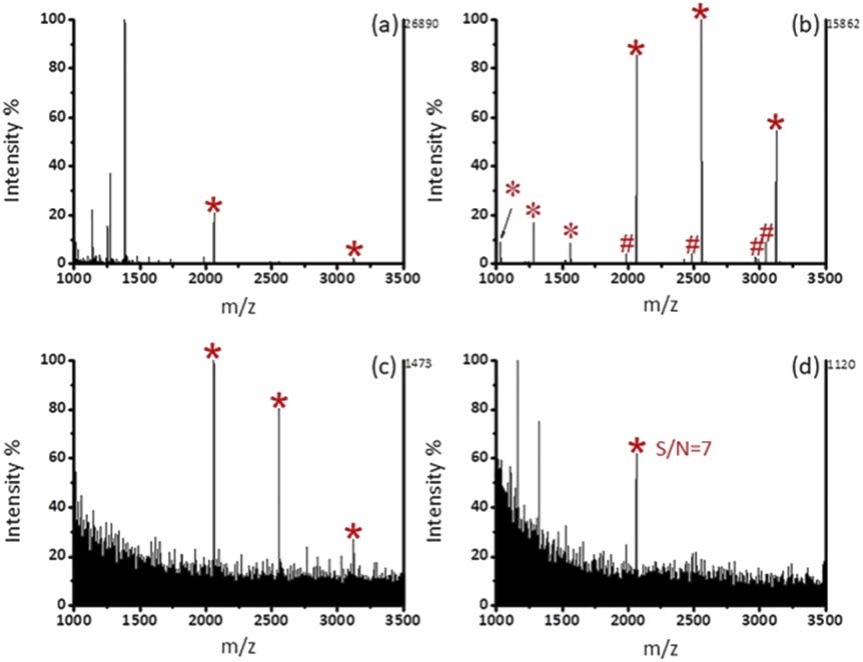
MALDI-TOF MS spectra of tryptic digest of β-casein. (a) 10 fmol without enrichment, (b) 10 fmol, (c) 0.1 fmol, and (d) 0.08 fmol after enrichment by Fe_3_O_4_@mSiO_2_-PFIL-Ti^4+^ nanoparticles. Phosphopeptides are labeled as *, dephosphopeptides are labeled as #, and doubly charged ion peaks are labeled as *.

As shown in Fig. 3b, after enrichment with Fe_3_O_4_@mSiO_2_-PFIL-Ti^4+^, 3 phosphopeptide signals (*m*/*z* 2061, 2556, and 3122), 4 phosphopeptide dephosphorylated fragments and 3 doubly charged ion peaks (*m*/*z* 1031, 1278, and 1561) were observed. The results show that Fe_3_O_4_@mSiO_2_-PFIL-Ti^4+^ nanoparticles can enrich phosphopeptides specifically. In order to further evaluate the properties of enrichment, we studied sensitivity of this nanoparticles. Three signals of phosphopeptides with high S/N ratios (*m*/*z* 2061, 2556, and 3122) remained clearly visible (Fig. 3c), even though the amount of β-casein digestion was reduced to 0.1 fmol. Moreover, when the amount of the digested β-casein was reduced to 0.08 fmol, one phosphopeptide peak was still observed (S/N = 7) (Fig. 3d). The low detection limit can be attributed to the combined advantages of flexible linkers, strong polydentate phosphonate chelators and high loading of metal ions. The results indicated that Fe_3_O_4_@mSiO_2_-PFIL-Ti^4+^ showed the excellent ability for phosphopeptides enrichment, which provides basis for further evaluation of enrichment performance.

**Fig. 4:**
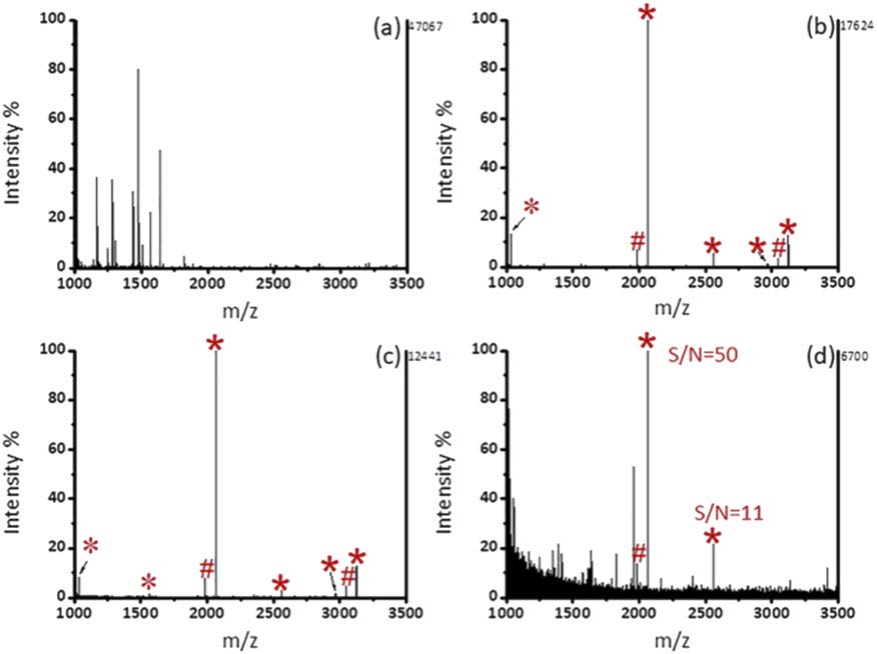
MALDI-TOF MS spectra of a digest mixture of β-casein (1.43 pmol) and BSA. (a) 1 : 1000 (β-casein/BSA, molar ratio) without enrichment, (b) 1 : 1000, (c) 1 : 5000, and (d) 1 : 10 000 after enrichment by Fe_3_O_4_@mSiO_2_-PFIL-Ti^4+^ nanoparticles. Phosphopeptides are labeled as *, dephosphopeptides are labeled as #, and the double charge ion peaks are labeled as *.

We then investigated the enrichment selectivity of Fe_3_O_4_@mSiO_2_-PFIL-Ti^4+^ nanoparticles to phosphopeptides from trypsin digested mixtures of β-casein and BSA. No signals of phosphopeptides were seen without enrichment, as revealed in Fig. 4a. After treatment with Fe_3_O_4_@mSiO_2_-PFIL-Ti^4+^ nanoparticles, 4 phosphopeptides (*m*/*z* 2061, 2556, 2966, and 3122) with strong intensity were identified for two samples diluted to 1000 and 5000 times by BSA digestion solution, respectively (Fig. 4b and c). Two phosphorylated peptides (*m*/*z* 2061 and 2556) with appreciable S/N ratio (50 and 11, respectively) were still detectable (Fig. 4d), even though β-casein digestion was diluted with 10 000-fold BSA digestion, demonstrating the super specificity and selectivity of Fe_3_O_4_@mSiO_2_-PFIL-Ti^4+^ nanoparticles.

**Fig. 5:**
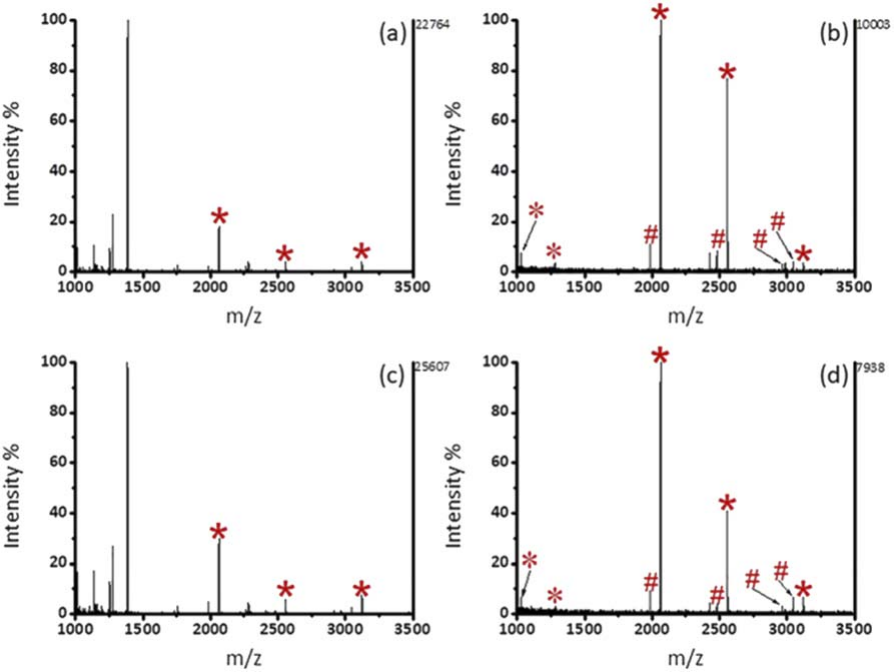
MALDI-TOF MS spectra of two kinds of mixtures with different mass ratios (β-casein kept at 2 pmol). 1 : 2000 (β-casein digest/BSA protein) (a) without enrichment and (b) after enrichment by Fe_3_O_4_@mSiO_2_-PFIL-Ti^4+^ nanoparticles; 1 : 2000 : 2000 (β-casein digest/BSA/β-casein) (c) without enrichment and (d) after enrichment by Fe_3_O_4_@mSiO_2_-PFIL-Ti^4+^ nanoparticles. Phosphopeptides are labeled as *, dephosphopeptides are labeled as #, and the double charge ion peaks are labeled as *.

In addition, the size-exclusion effect of Fe_3_O_4_@mSiO_2_-PFIL-Ti^4+^ nanoparticles was also investigated. Two kinds of mixtures with different mass ratios of β-casein digest and BSA protein (1 : 2000), and β-casein digest, BSA protein and β-casein protein (1 : 2000 : 2000) were configured to simulate real bio-samples. As shown in Fig. 5, the signal peaks of phosphopeptides in high intensities were detected, indicating that the interference of large proteins was eliminated after the procedure of enrichment. Due to the small pore size (3.13 nm) of the material, nonphosphorylated proteins (BSA) and phosphorylated proteins (β-casein) with sizes of about 8 nm and 12 nm can be excluded.^27,28^

In order to achieve large-scale commercialization of materials, it is necessary to evaluate the recyclability and batch-to-batch repeatability of materials. Thus, we first examined the recyclability of Fe_3_O_4_@mSiO_2_-PFIL-Ti^4+^ nanoparticles in enriching β-casein digest (2 pmol). The MS spectra shown in Fig. S5 (ESI†) demonstrated that the result of the sixth time is almost the same as that of the first time. Two mono-phosphopeptides (2061 and 2556) and two multi-phosphopeptides (*m*/*z* 2966 and 3122) were present with high intensities in mass spectra. After 12 times of reuse, the same four phosphorylated peptides can still be observed with strong intensities. The facts described above illustrate that due to the strong binding ability of multidentate phosphonate ligands to metal ions, the as-synthesized Fe_3_O_4_@mSiO_2_-PFIL-Ti^4+^ nanoparticles are robust and can be used several times without serious loss of enrichment ability. Additionally, nanoparticles from different batches were used to enrich phosphopeptides for the evaluation of batch-to-batch repeatability of Fe_3_O_4_@mSiO_2_-PFIL-Ti^4+^ nanoparticles. Fig. S6 (ESI†) showed that there was no significant difference in the enrichment results of the two batches of samples. These results prove that the method of preparing Fe_3_O_4_@mSiO_2_-PFIL-Ti^4+^ nanoparticles is feasible.

### Enrichment of phosphopeptides from bio-samples

All experiments were performed in accordance with the guidelines of declaration of Helsinki, and approved by the ethics committee at Ningbo University. Informed consents were obtained from human participants of this study. Firstly, the enrichment specificity of Fe_3_O_4_@mSiO_2_-PFIL-Ti^4+^ to endogenous phosphopeptides was evaluated by using human serum as a sample. Before enrichment, there were only two signal peaks of phosphopeptides in the whole MS spectrum, which were almost occupied by non-phosphopeptides as shown in Fig. S7a (ESI†). After enrichment, four typical peaks of phosphopeptides with strong intensities were identified. The detailed information of identified phosphopeptides in human serum was listed in the Table S2 (ESI†). These results indicate that Fe_3_O_4_@mSiO_2_-PFIL-Ti^4+^ nanoparticles have the potentiality to enrich phosphopeptides from complex bio-samples.

**Fig. 6:**
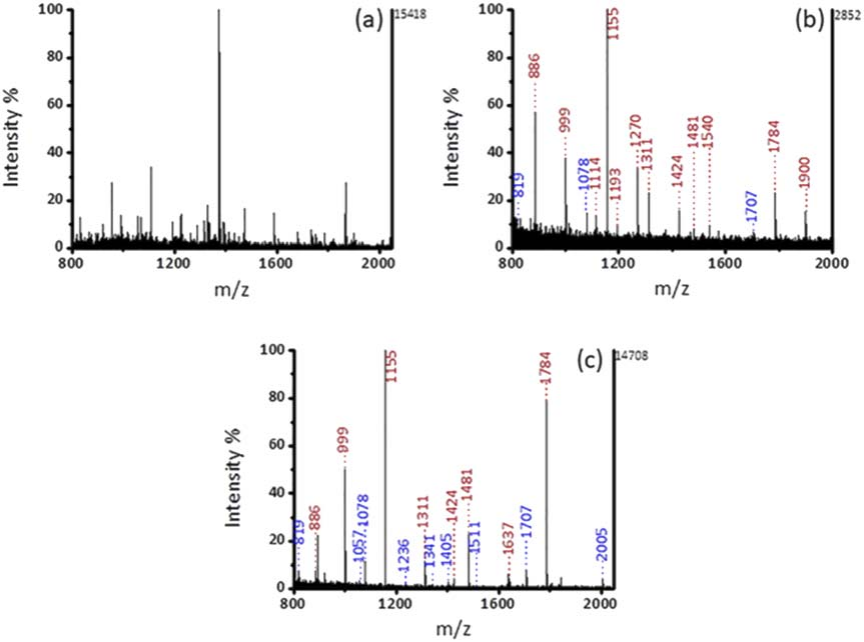
MALDI-TOF MS spectra of human saliva (a) without enrichment, after enrichment (b) by commercial PurMag Si–TiO_2_, and (c) by Fe_3_O_4_@mSiO_2_-PFIL-Ti^4+^ nanoparticles. The mono- and multi-phosphopeptides were labeled as blue and red font, respectively.

Saliva with accessibility and noninvasive availability is often utilized as a representative of body fluids, because biomarkers present in saliva can response the health status of the body.^29,30^ Signal peaks of non-phosphopeptides dominated the MS spectrum without enrichment in Fig. 6a. Compared with commercial PurMag Si–TiO_2_, which enriched 15 endogenous phosphopeptides including 3 mono-phosphopeptides and 12 multi-phosphopeptides (Fig. 6b), after enrichment by Fe_3_O_4_@mSiO_2_-PFIL-Ti^4+^ (Fig. 6c), 17 endogenous phosphopeptides including 9 mono-phosphopeptides and 8 multi-phosphopeptides were discerned with stronger intensities and cleaner background. This result verified that mesoporous structure with size-exclusion effect has the advantage on the exclusion of large proteins again. Moreover, the materials had no preference for mono-phosphopeptides or multi-phosphopeptides. The detailed information of the enriched phosphopeptides from human saliva is exhibited in ESI (Table S3†). Additionally, the enrichment performance of Fe_3_O_4_@mSiO_2_-PFIL-Ti^4+^ nanoparticles is comparable to those of the reported magnetic materials (Table 1). Therefore, the above results further prove the great application potentiality of the synthesized nanoparticles in enriching phosphopeptides from complex bio-samples.

**Table 1:** Comparison of our material Fe_3_O_4_@mSiO_2_-PFIL-Ti^4+^ with the recently reported adsorbent materials

Affinity materials	Sensitivity β-casein (fmol μL^−1^)	Selectivity β-casein/BSA (molar ratio)	The number of phosphopeptides from saliva (mono-/multi-)	Ref.
Fe_3_O_4_@mSiO_2_-PO_3_-Ti^4+^/Zr^4+^	1	1 : 500 (mass ratio)	25	31
Ti^4+^-MGMSs	0.5	1 : 500 (mass ratio)	14 (4/10)	32
Fe_3_O_4_@PDA@Zr-Ti-MOF	0.04	—	25 (16/9)	33
magG@PDA-Sn^4+^	8 (fmol)	1 : 1000	20 (8/12)	34
TiO_2_@SiO_2_-B(OH)_2_@Fe_3_O_4_@TiO_2_	0.8	1 : 1000	12	35
Fe_3_O_4_@mSiO_2_-Ti^4+^	0.1	—	13	36
Fe_3_O_4_@mSiO_2_@Ti^4+^-Zr^4+^	0.1	—	13 (11/2)	37
Fe_3_O_4_@TiO_2_-ZrO_2_@mSiO_2_	0.2	—	14 (3/11)	38
4μ-PEO-Ti^4+^	2 (fmol)	1 : 1000	15 (1/14)	39
Fe_3_O_4_ MNCs affinity probe	—	—	11	40
Fe_3_O_4_@mSiO_2_-PFIL-Ti^4+^	0.08 (fmol)	1 : 10 000	17 (9/8)	Our work

## Conclusions

In summary, we prepared a new magnetic IMAC nanoparticles with PFIL as the surface modifier. Such IMAC nanoparticles have strong hydrophilicity with flexible linkers and strong binding phosphonate chelators, small steric resistance with large specific surface area and rapid magnetic response. This material shows excellent sensitivity, selectivity and size exclusion ability, as well as outstanding recyclability in phosphopeptides enrichment. Hence it is possible that the new magnetic IMAC nanomaterial will be potential for the study of phosphoproteomics.

## Author contributions

Yufei Jiang: investigation, formal analysis, validation, visualization, data curation, and writing – original draft. Weida Liang: investigation and writing – review & editing. Binbin Wang: investigation and formal analysis. Quanshou Feng: investigation. Chenglong Xia: formal analysis. Qiyao Wang: investigation, formal analysis. Yinghua Yan: investigation. Lingling Zhao: formal analysis. Wei Cui: conceptualization, formal analysis, methodology, funding acquisition, supervision, writing – original draft, and writing – review & editing. Hongze Liang: conceptualization, formal analysis, methodology, funding acquisition, supervision, writing – original draft, and writing – review & editing.

## Conflicts of interest

There are no conflicts to declare.

## Supplementary Material

RA-012-D2RA04609A-s001
