# Supplementary material for: Magnetic mesoporous silica nanoparticles modified by phosphonate functionalized ionic liquid for selective enrichment of phosphopeptides
Source: RSC Adv. 2022 Sep 21;12(41):26859–65. doi: 10.1039/d2ra04609a (PMC9490807; doi:10.1039/d2ra04609a)
Supplement: RA-012-D2RA04609A-s001 [file RA-012-D2RA04609A-s001.pdf]

Electronic Supplementary Material (ESI) for RSC Advances.
This journal is © The Royal Society of Chemistry 2022

Supporting Information

Magnetic Mesoporous Silica Nanoparticles Modified by Phosphonate Functionalized Ionic Liquid for Selective Enrichment of Phosphopeptides

Yufei Jiang,[a] Weida Liang,[a] Binbin Wang,[a] Quanshou Feng,[a] Chenglong Xia,[a] Qiyao Wang,[b] Yinghua Yan,[a] Lingling Zhao,[a] Wei Cui*[b] and Hongze Liang*[a]

[a] *Key Laboratory of Advanced Mass Spectrometry and Molecular Analysis of Zhejiang Province, School of Materials Science and Chemical Engineering, Ningbo University, Ningbo 315211, China*

[b] *Ningbo Key Laboratory of Behavior Neuroscience, Zhejiang Province Key Laboratory of Pathophysiology, School of Medicine, Ningbo University, Ningbo 315211, China*

* Corresponding authors.

E-mail addresses: lianghongze@nbu.edu.cn (H. Liang), cuiwei@nbu.edu.cn (W. Cui)

Contents

Materials and reagents 3
Characterization of the nanoparticles 3
Synthesis of Fe_3O_4@$mSiO_2$-PFIL-Ti^{4+} 4
Sample preparation 5
Enrichment of Phosphopeptides 6
MALDI-TOF MS Analysis 7
Figures S1-S7 8
Tables S1–S3 12
References 13

Materials and reagents

$FeCl_3 \cdot 6H_2O$ and ethylene glycol were acquired from Macklin (Shanghai, China). Cetyltrimethyl ammonium bromide (CTAB) was obtained from Beijing Solarbio Science & Technology. Tetraethyl orthosilicate (TEOS), ammonia solution (25-28 wt%), sodium acetate, acetonitrile (ACN), titanium sulfate ($Ti(SO_4)_2$) and sodium hydroxide were obtained from Sinopharm Chemical Reagent Co., Ltd. Triethyl phosphite was acquired from Chengdu Gracia Chemical Technology. Pyridoxal 5'-phosphate was obtained from Shanghai Yuanye Biotechnology. 1,3-dibromopropane was obtained from HWRK Chem. Trifluoroacetic acid (TFA) was purchased from Aladdin. 3-aminopropyltriethoxysilane (APTES) was purchased from Adamas-beta. PuriMag Si-TiO_2 was obtained from PuriMag Biotechnology Ltd. β-casein, iodoacetamide (IAA), dithiothreitol (DTT) and BSA as well as 2,5-dihydroxybenzoic acid (DHB) were all obtained from Sigma Aldrich.

Human serum and saliva samples were collected from healthy a volunteer, on the basis of the protocol approved by Ningbo University Ethical Board.

Characterization of the nanoparticles

Scanning electron microscopy (SEM) images and energy dispersive X-ray analysis (EDX) were recorded on a Nova NanoSEM 450 electron microscope (FEI, USA). Transmission electron microscopy (TEM) images were carried out on a Tecnai F20 electron microscope (FEI, USA). Fourier-transform infrared spectroscopy (FT-IR) was taken on a NICOLET 6700 Fourier transform infrared spectrometer (Thermo Scientific, USA) using KBr pellets. The X-ray photoelectron spectra (XPS) were performed by a Thermo Scientific K-Alpha spectrometer (Thermo Scientific, USA). Inductively coupled plasma optical emission spectroscopy (ICP-OES) was analyzed on an Aglient 5110 (Aglient, USA). The zeta potentials and nanoparticle sizes were measured on a Zetasizer Nano ZS90 analyzer (Malvern, UK). Nitrogen adsorption-desorption isotherms were measured on an ASAP2020 analyzer (Micromeritics,

USA). The Brunauer-Emmet-Teller (BET) method was employed to calculate the specific surface areas, and the pore volumes and pore size distributions were obtained by the Barrett-Joyner-Halenda (BJH) method. Water contact angles were measured on a Dataphysics OCA25 with 5.0 μL water drop. UV-Vis spectra were recorded on a UV-2600 spectrophotometer (Shimadzu, Japan). The magnetic properties were measured by Lakeshore 7410 vibrating sample magnetometer (VSM) (Lakeshore, USA) at room temperature.

Synthesis of Fe_3O_4@$mSiO_2$-PFIL-Ti^{4+}

Magnetic particles were prepared by solvothermal reaction as described by Deng et al.[1] The core-shell Fe_3O_4@$mSiO_2$ microspheres were prepared according to the previously reported method with modification.[2] The mixture of 1000 mg CTAB, 100 mL deionized water and 100 mg Fe_3O_4 was treated with ultrasound for 30 min. The suspension was ultrasonicated for another 10 min after 800 mL water and 100 mL NaOH aqua (0.01 M) were added in above mixture in turn. Subsequently, mechanical stirring was used in the mix at 60 °C for 30 min. Finally, 5 mL uniform mixture of TEOS and ethanol (v/v = 1:4) was dripped continuously into the container and reacted for 14 h at 60 °C. The solid was rinsed to neutral with water and then washed with ethanol for 4 times. This material was dried at 85 °C for 5 h later. To remove the template CTAB, 60 mg purified microspheres were re-dispersed in 80 mL of methanol and 0.5 mL of 37% (w/w) hydrochloric acid. After that, the liquid mixture was mechanically agitated and refluxed at 60 °C for one day. Finally, Fe_3O_4@$mSiO_2$ nanoparticles were produced successfully after the nanoparticles were washed with deionized water and ethanol.

Preparations of Fe_3O_4@$mSiO_2$-NH_2, Fe_3O_4@$mSiO_2$-PFILOEt and Fe_3O_4@$mSiO_2$-PFIL-Ti^{4+} were referred to the previously reported literature.[3]

The surface modification of Fe_3O_4@$mSiO_2$ nanoparticles was carried out using APTES to afford Fe_3O_4@$mSiO_2$-NH_2 nanoparticles. APTES was used as the silylation agent. 100 mg of Fe_3O_4@$mSiO_2$ and 30 mL of toluene were ultrasonically

dispersed for 15 min. Afterwards, 1 mL APTES was added to the suspension and the mixture was reacted for 12 h by mechanically stirring at 110 ℃. The resultant Fe_3O_4@mSiO_2-NH_2 was collected with a magnet, then rinsed thoroughly with ethanol and water, and finally dried at 85 ℃.

After 100 mg of Fe_3O_4@mSiO_2-NH_2 and 60 mL of toluene were mixed together, the mixture was ultrasonically dispersed for 0.5 h. Subsequently, 200 mg diethyl (3-bromopropyl) phosphonate was added to the suspension. The mixture was mechanically agitated at 85 ℃ overnight. The obtained nanoparticles (denoted as Fe_3O_4@mSiO_2-PFILOEt) were assembled by magnetic separation and the redundant diethyl (3-bromopropyl) phosphonate was removed by washing with ethanol.

Fe_3O_4@mSiO_2-PFILOEt (80 mg) was hydrolyzed by 510 μL $Si(CH_3)_3Br$. After collected by magnet, material was washed with sodium hydroxide aqueous solution (pH = 11) to neutral to obtain Fe_3O_4@mSiO_2-PFIL-Na^+. Particles were gathered with a magnetic field and then washed with ethanol and water and dried at 85 ℃ under vacuum.

Finally, 100 mg Fe_3O_4@mSiO_2-PFIL-Na^+ was incubated in 30 mL $Ti(SO_4)_2$ aqueous solution (0.024 g·mL^{-1}) at 25 ℃ for 1 h to immobilize Ti^{4+}. The resulting Fe_3O_4@mSiO_2-PFIL-Ti^{4+} were assembled with a magnet and washed with water. The procedure was repeated several times and the product was dried out in vacuum at 85 ℃ overnight.

Sample preparation

β-casein (5 mg) was dissolved in 1 mL NH_4HCO_3 buffer (25 mM, pH = 8), and incubated with trypsin (1/50, w/w) at 37 °C for 16 h. The mixture obtained was stored at -20 °C for further use.

BSA (1 mg) was dissolved in 0.1 mL NH_4HCO_3 buffer (50 mM, containing 8 M urea), and the mixture was incubated with 0.2 mL DTT solution (0.1 M) for 30 min at 37 °C, then adding 0.2 mL IAA solution (0.2 M) and reacting in dark at room temperature for 30 min. After that, the mixture was diluted to 1.0 mL with NH_4HCO_3

buffer (50 mM), and incubated with trypsin (1/50, w/w) at 37 °C for 16 h. The digested product (5 mg·mL^{-1}) was stored at -20 °C. For the study on selectivity, a series of samples were prepared as follows. Each digest of β-casein (kept at 1.43 pmol) was diluted by varying the amount of digested BSA to 1:1000, 1:5000 and 1:10000 (molar ratio), respectively. For the study on size-exclusion effect, each digest of β-casein (kept at 2 pmol) was diluted by addition of BSA to reach mass ratio 1:2000, or by addition of β-casein and BSA to reach mass ratio 1:2000:2000, respectively.

Human serum (2.5 μL) was mixed with loading buffer (22.5 μL) and centrifuged. The supernatant was collected and stored at -20 °C.

Human saliva (12.5 μL) was mixed with 0.2% TFA in equal volume and centrifuged for 10 min, then the supernatant was collected and stored at -20 °C.

Enrichment of Phosphopeptides

0.25 mg of Fe_3O_4@mSiO_2-PFIL-Ti^{4+} nanoparticles and 100 μL loading buffer which contains 1 μL β-casein digests were mixed, and vibrated. By employing a magnet, the supernatant was easily and quickly removed, and the collection of nanocomposites was washed with loading buffer thoroughly before addition of 10 μL of NH_4OH solution to release the captured phosphopeptides. Following analysis was conducted on a MALDI-TOF MS.

The process of enriching phosphopeptides from mixtures (the digested mixtures of β-casein and BSA; digested β-casein, β-casein and BSA; digested β-casein and BSA) was carried on, just as previously described. For the sake of evaluating the reusability of Fe_3O_4@mSiO_2-PFIL-Ti^{4+} nanoparticles, same amount of β-casein digest (2 pmol) was used to enrich.

Procedure of enriching endogenous phosphopeptides from biological samples, human serum and saliva, was same as the above mentioned excluding the change that 25 μL of pretreated serum or saliva were used, respectively.

MALDI-TOF MS Analysis

0.5 μL sample mixed with 0.5 μL of DHB solution (20 mg·mL^{-1}, 50% ACN/ 49% H_2O/ 1% H_3PO_4, v/v/v) was dropped upon the plate and dried, and analyzed by MALDI-TOF MS. MALDI-TOF spectrometer autoflex maX (Bruker Scientific, USA) was used in positive ion mode with an Nd:YAG laser (383 nm). The acceleration voltage was run at 20 kV, and the repetition rate was 1000 Hz.

Figures S1-S7

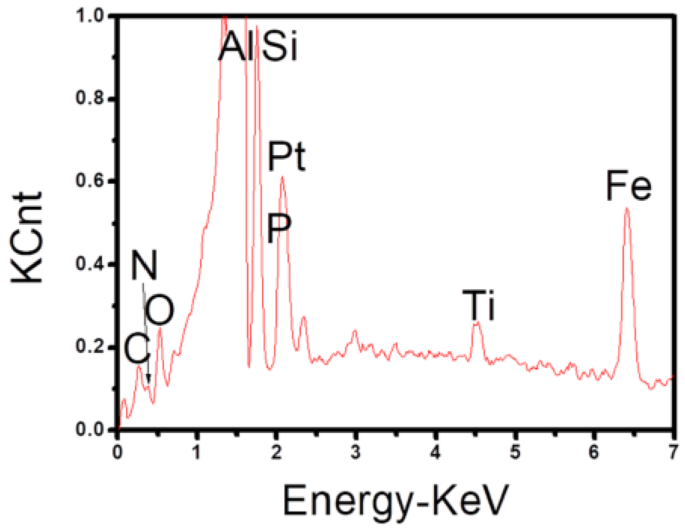

Fig. S1 EDX image of Fe_3O_4@mSiO_2-PFIL-Ti^{4+}.

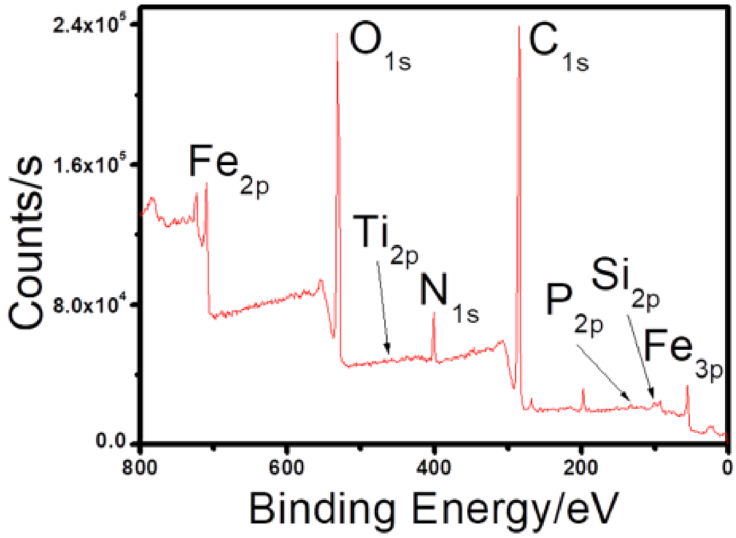

Fig. S2 XPS spectrum of Fe_3O_4@mSiO_2-PFIL-Ti^{4+}.

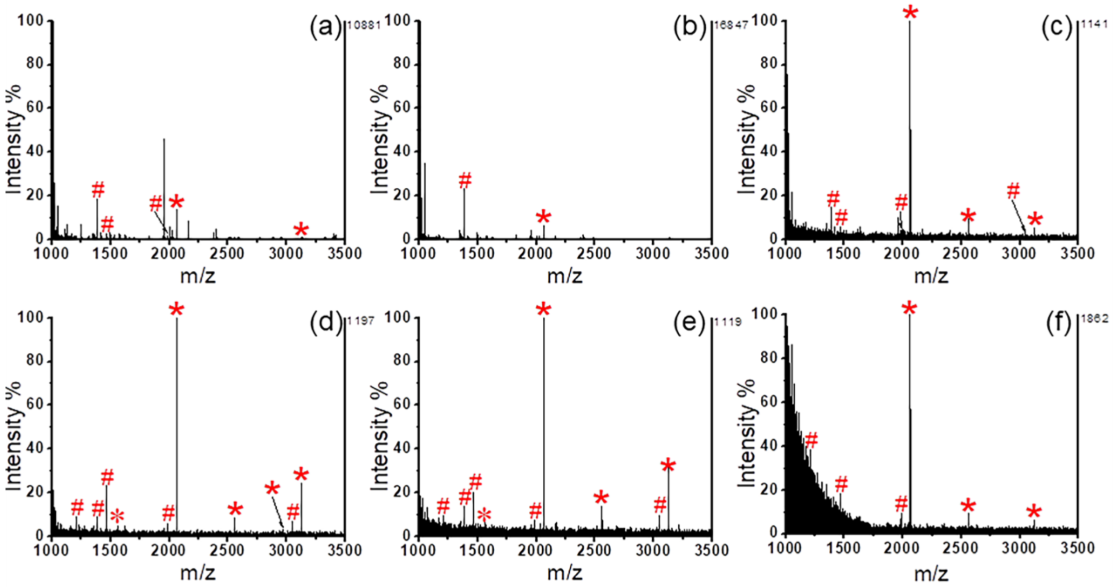

Fig. S3 The MALDI-TOF MS spectra of tryptic digest mixture of β-casein and BSA (1/100, molar ratio) with different loading buffers containing 50% ACN and (a) 0.1%TFA, (b) 0.2%TFA, (c) 0.5%TFA, (d) 1%TFA, (e) 2%TFA, (f) 3%TFA. Phosphopeptides are labeled as *, dephosphopeptides are labeled as #, and the double charge ion peaks are labeled as *.

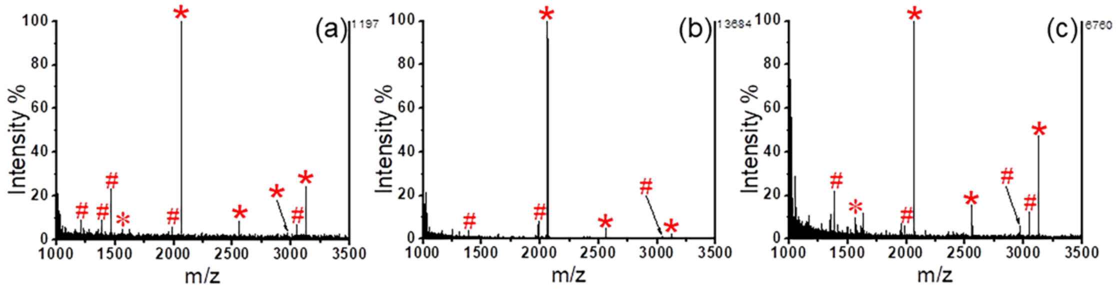

Fig. S4 The MALDI-TOF MS spectra of tryptic digest mixture of β-casein and BSA (1/100, molar ratio) with different elution buffer of NH_4OH diluted with deionized water (a) 3 v%, (b) 5 v% and (c) 10 v%. Phosphopeptides are labeled as *, dephosphopeptides are labeled as #, and the double charge ion peaks are labeled as *.

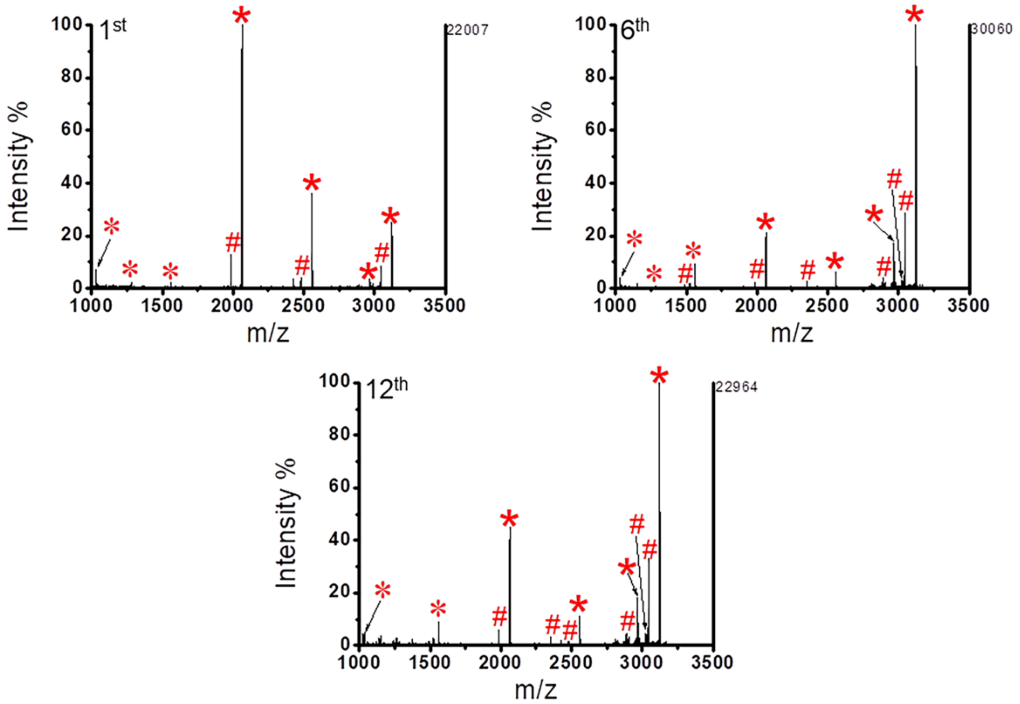

Fig. S5 MALDI-TOF MS spectra of β-casein tryptic digest (2 pmol) after enrichment by Fe_3O_4@mSiO_2-PFIL-Ti^{4+} nanoparticles for the 1st, 6th and the 12th time. Phosphopeptides are labeled as *, dephosphopeptides are labeled as #, and the double charge ion peaks are labeled as *.

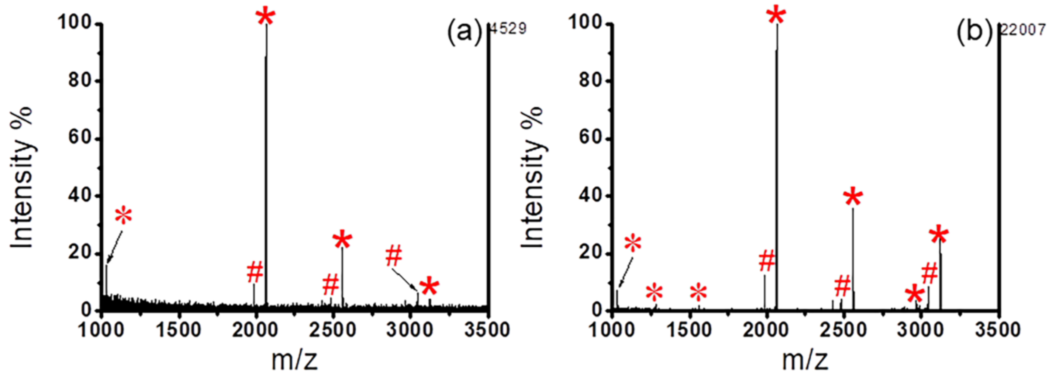

Fig. S6 MALDI-TOF MS spectra of β-casein tryptic digest after enrichment by different batches Fe_3O_4@mSiO_2-PFIL-Ti^{4+} nanoparticles (a) batch I and (b) batch II. Phosphopeptides are labeled as *, dephosphopeptides are labeled as #, and the double charge ion peaks are labeled as *.

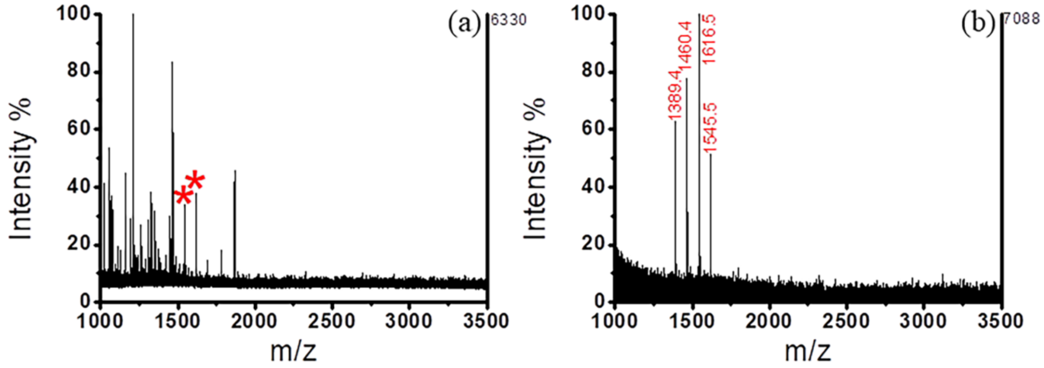

Fig. S7 MALDI-TOF MS spectra of human serum (a) before and (b) after enrichment by Fe_3O_4@mSiO_2-PFIL-Ti^{4+} nanoparticles

Tables S1–S3

Table S1. The detailed information of phosphopeptides from β-casein digests enriched by Fe_3O_4@$mSiO_2$-PFIL-Ti^{4+}. $\underline{S}$: phosphorylation site, a: doubly charged ion (β-casein tryptic digest).

No	m/z	Peptide Sequence	Phosphorylation Site
1	1031[a]	FQ$\underline{S}$EEQQQTEDELQDK	1
2	1278[a]	FQ$\underline{S}$EEQQQTEDELQDKIHPF	1
3	1561[a]	RELEELNVPGEIVE$\underline{S}$L$\underline{SSS}$EESITR	4
4	2061	FQ$\underline{S}$EEQQQTEDELQDK	1
5	2556	FQ$\underline{S}$EEQQQTEDELQDKIHPF	1
6	2966	ELEELNVPGEIVE$\underline{S}$L$\underline{SSS}$EESITR	4
7	3122	RELEELNVPGEIVE$\underline{S}$L$\underline{SSS}$EESITR	4

Table S2. The detailed information of phosphopeptides identified from human serum. $\underline{S}$: phosphorylation site.

No.	m/z	Peptide Sequence	Phosphorylation Site
1	1390	D$\underline{S}$GEGDFLAEGGGV	1
2	1459	AD$\underline{S}$GEGDFLAEGGGV	1
3	1546	D$\underline{S}$GEGDFLAEGGGVR	1
4	1616	AD$\underline{S}$GEGDFLAEGGGVR	1

Table S3. The detailed information of phosphopeptides from human saliva enriched by Fe_3O_4@$mSiO_2$-PFIL-Ti^{4+}. $\underline{S}$: phosphorylation site.

No.	[MH$^+$ Da]	Sequence	Fe_3O_4@$mSiO_2$-PFIL-Ti^{4+}	PuriMag Si-TiO_2	Ref
1	819	DGyMPR	√	√	4
2	886	KLsEATR	√	√	4
3	999	ssEEKFL	√	√	5
4	1057	VsQEESPSL	√		6
5	1078	sSEEKFLR	√	√	7
6	1114	tDsDEKFI		√	4
7	1155	ssEEKFLR	√	√	5
8	1193	DSsEEKFLR		√	7
9	1236	sEQFLDEER	√		5
10	1270	DssEEKFLR		√	4
11	1311	ssEEKFLRR	√	√	5
12	1341	DsHEKRHHGY	√		5
13	1405	GDsEQFLDEER	√		7
14	1424	DssEEKFLRR	√	√	5

15	1481	ssEEKFLRRIG	√	√	5
16	1511	DVPLVISDGGDsEQ	√		7
17	1540	SccLIPQTVsHR		√	8
18	1637	ssEEKFLRRIGR	√		7
19	1707	PGKPQGPPPQGGNQsQG	√	√	9
20	1784	ISDGGDsEQFIDEER	√	√	10
21	1900	ISDGGDsEQFLDEERQ		√	4
22	2005	VISDGGDsEQFIDEERQ	√		10

References

1. Y. Deng, D. Qi, C. Deng, X. Zhang and D. Zhao, *J Am Chem Soc*, 2008, **130**, 28-29.
2. X. W. Fang, J. Z. Yao, X. F. Hu, Y. Li, G. Q. Yan, H. Wu and C. H. Deng, *Talanta*, 2020, **207**, 120313.
3. B. B. Wang, W. D. Liang, H. Z. Liang, B. Liu, Q. Y. Wang, Y. H. Yan, T. T. Wang, Y. F. Jiang, Y. L. Jiang, H. D. Qiu, L. H. Lu, W. Cui, Y. Zhou, L. L. Zhao and H. B. Yu, *Acs Sustain Chem Eng*, 2021, **9**, 7930-7940.
4. J. W. Wang, Z. D. Wang, N. R. Sun and C. H. Deng, *Microchim Acta*, 2019, **186**, 236.
5. Q. Liu, N. Sun, M. Gao and C.-h. Deng, *ACS Sustainable Chemistry & Engineering*, 2018, **6**, 4382-4389.
6. X. F. Hu, Y. L. Li, A. Z. Miao and C. M. Deng, *Microchim Acta*, 2020, **187**, 400.
7. H. Z. Lin and C. H. Deng, *Proteomics*, 2016, **16**, 2733-2741.
8. Y. L. Li, L. L. Liu, H. Wu and C. H. Deng, *Anal Chim Acta*, 2019, **1079**, 111-119.
9. N. R. Sun, J. W. Wang, J. Z. Yao, H. M. Chen and C. H. Deng, *Microchim Acta*, 2019, **186**, 159.
10. N. R. Sun, C. H. Deng, Y. Li and X. M. Zhang, *Acs Appl Mater Inter*, 2014, **6**, 11799-11804.
